# Can advancing environmental justice safeguard older adults' health? — Evidence from China's establishment of environmental courts

**DOI:** 10.3389/fpubh.2025.1599907

**Published:** 2025-08-27

**Authors:** Shubao Jing, Rongzhe Zhang, Chan Du, Yuting Liu, Yikun Qing, Liang Chen, Min He, Wei Peng, Jiling Ye

**Affiliations:** ^1^School of International Relations, Sichuan University, Chengdu, Sichuan, China; ^2^School of Business, Chengdu University, Chengdu, Sichuan, China; ^3^Journal of Social Science Research, Sichuan Academy of Social Sciences, Chengdu, Sichuan, China; ^4^School of Environment, Sichuan Agricultural University, Chengdu, Sichuan, China; ^5^College of Social Science, Duke Kunshan University, Kunshan, Jiangsu, China; ^6^School of Finance and Accounting, Chengdu Jincheng College, Chengdu, Sichuan, China; ^7^Chengdu Eighth People's Hospital (Geriatric Hospital of Chengdu Medical College), Sichuan University, Chengdu, China; ^8^West China School of Public Health and West China Fourth Hospital, Sichuan University, Chengdu, Sichuan, China

**Keywords:** environmental courts, environmental justice, environmental regulation, older adults' health, Difference-in-Differences (DID) models

## Abstract

**Introduction:**

Amidst global population aging, enhancing the health and well-being of older adults has become a critical concern. The role of environmental justice in influencing older adults' health, however, remains underexplored.

**Methods:**

Utilizing five waves of data from the China Health and Retirement Longitudinal Study (CHARLS) between 2011 and 2020, this study examines the impact of environmental courts on older adults' health using a multi-period Diference-in-Diferences (DID) model.

**Results:**

Results show that environmental tribunals significantly improved older adults' health, with more pronounced benefits for individuals in cities along the Yangtze and Yellow River Basins, those with lower educational attainment, and residents of non-resource-based cities. Mechanism analyses indicate that stricter enforcement of environmental penalties, improved water quality, and reduced air pollution are key pathways driving these improvements.

**Discussion:**

Policy recommendations include expanding the establishment of environmental courts nationwide, implementing targeted and region-specific policies, increasing public awareness about environmental courts, and enhancing mechanisms for policy evaluation and feedback. These measures aim to foster a synergistic relationship between environmental governance and public health improvement.

## 1 Introduction

The world is facing a clear trend of population aging (1). The share of the population aged 60 and above will increase from 1 billion in 2020 to 1.4 billion in 2030, accounting for 16.7% of the global population, and this figure is expected to double by 2050. Among them, China is particularly serious. It is estimated that by the end of 2025, the population of China aged 60 or above will exceed 300 million, accounting for more than 20% of the population. By 2034, this number may rise to more than 400 million, accounting for 30% of the population (2). The increase in the age of the world population is accompanied by an increase in the incidence of various aging-related diseases (3). The older adults are one of the most vulnerable groups (4), and the vulnerability of the older adults brings the risk of various infections and reduces all forms of immune response (5). In addition, aging is a major driver of most chronic diseases. It can be seen that compared with young people, the older adults face more serious health problems (6). Actively responding to population aging and improving the health of the older adults has become the national strategy of every country.

China's economic prosperity has led to serious environmental pollution and brought significant health risks to residents (7). According to the experience of ecological and environmental governance around the world, the construction of the rule of law is an institutional guarantee to ensure that other environmental regulation methods can play an effective role (8). Since the promulgation of the “Environmental Protection Law of the People's Republic of China (Trial)” in 1979, China's environmental protection legal system has been continuously improved. However, due to the lack of a dedicated environmental judiciary, the existing environmental legal system has not achieved significant results in practice. Therefore, in order to substantially resolve local environmental problems, environmental justice, and environmental legislation need to cooperate. In November 2007, Guiyang City established China's first environmental protection court. The Supreme People's Court established a special environmental court in 2014, representing the improvement of the environmental court system, which is an important part of China's environmental governance. The Environmental Protection Court is responsible for hearing criminal, civil, and administrative cases and enforcing claims related to environmental resources. It is a particularly useful institutional tool to resolve China's environmental dilemma (9). At present, China's environmental courts are the largest, the most comprehensive, and the types are quite diverse. As of the end of 2020, China has established 1,993 specialized judicial institutions for environmental resources, including 617 courts, 1,167 collegial panels, 209 people's courts and circuit courts, forming a relatively complete judicial organization system for professional environmental justice. According to the “China Environmental and Resources Trial (2023)”: as of 2023, people's courts at all levels across the country have fairly tried various environmental and resource cases in accordance with the law, and have accepted a total of 258,555 first-instance environmental and resource cases and concluded 231,830 (10).

The establishment of environmental protection courts has strengthened environmental justice, improved administrative law enforcement by local governments, increased legal risks related to illegal emissions and the pressure on polluters by public opinion, played an important role in pollution control, and provided new ideas for improving the health status of the older adults (11, 12). Economists focus primarily on evaluating the economic and environmental benefits of establishing environmental courts. Although the positive impact of environmental regulation on public health has been widely confirmed, existing research focuses on command-control, market-oriented, and administrative tools, while lacking causal identification of the health consequences of judicial tools, such as environmental courts (13). As a group with physiological fragility, long accumulation of pollution exposure and low social and economic adaptability, the older adults should have the greatest health benefits, but they are rarely studied specifically. This paper uses quasi-natural experimental methods to systematically evaluate the impact of environmental justice on the health of the older adults in China for the first time, incorporate environmental justice into the framework of health benefits analysis, and expand the boundaries of the health effects of environmental regulation tools; provide empirical basis for how to optimize the health of the older adults through judicial means in the context of aging; use CHARLS microdata and the dual difference method (DID) to solve the causal identification problem of the health effects of judicial policies.

## 2 Literature review and institutional background

### 2.1 Literature review

The nexus between environmental pollution and human health has garnered significant academic attention, generating an extensive body of literature from environmental, medical, public health, and economic perspectives. Environmental pollution encompasses various dimensions, including air, soil, solid waste, and water contamination, with numerous studies highlighting their detrimental effects on physical health. For instance, air pollution is linked to increased morbidity and mortality from respiratory diseases, cardiovascular conditions, lung cancer, dementia (14, 15), and other ailments (16). Toxic metals from soil pollution can bioaccumulate in human tissues through the food chain, damaging the nervous, circulatory, and immune systems, leading to severe health issues (17, 18) Solid waste dumping fosters conditions conducive to the spread of diseases (19) and adversely affects respiratory health (20), causing a spectrum of respiratory illnesses, allergies, central nervous system disorders, and circulatory problems (21). Similarly, toxic heavy metals in water bodies are associated with cardiovascular diseases, cerebral thrombosis, and goiter, among other health challenges (22, 23).

In response to the multifaceted threats posed by environmental pollution, policymakers, and researchers have extensively explored environmental policies and interventions to mitigate its impact on public health. Environmental regulation has been widely recognized as a cornerstone for reducing environmental pollution levels while enhancing individual and social welfare (24, 25). These regulatory instruments encompass command-and-control measures, market-based mechanisms, and informal tools, which collectively form a comprehensive framework to promote sustainable environmental and health outcomes. Command-and-control environmental regulations involve mandatory legal provisions, technical standards, and restrictions aimed at mitigating pollution. For example, the Prevention and Control of Air Pollution Act (1987), targeting industrial and coal-fired emissions, and the creation of “two control zones” (1988) for acid rain and sulfur dioxide control provide institutional safeguards. Such measures have contributed to significant reductions in the prevalence of heart disease (26), endocrine disorders (27), urinary disorders (28), and obstetrics and gynecological diseases (29). Similarly, the pilot low-carbon city policies initiated in 2010 have reduced air pollution in pilot regions, leading to improved public health outcomes (30) and lower infant mortality rates (24). The Central Ecological Environment Protection Inspection System, launched in Hebei Province in 2016, has bolstered the efficacy of environmental supervision, established long-term mechanisms to curb pollution (31), and ultimately mitigated environmental degradation (32).

Market-based environmental regulation tools are market signals that enable polluters to make decisions about sewage disposal. The Interim Measures for the Collection of Sewage Charges issued in 1982 marked the formal establishment of the sewage charging system, which has been implemented nationwide since then. This was revised in 2003 with the implementation of the new Regulations on the Administration of Sewage Charges Collection and Use, which changed sewage charging from single-factor to multi-factor, and raised the standard for sewage charging. The sewage charges were changed from single-factor charges to multi-factor charges, and the standard of sewage charges was also increased (33). Studies indicate that these systems have successfully reduced emissions of SO_2_ and COD (34, 35), improved air quality, and decreased respiratory-related morbidity and mortality (36).

Informal environmental regulation tools engage the public as active participants in environmental conservation and protection. Initiatives such as the Environmental Impact Assessment Law (2003), Interim Measures on Public Participation in Environmental Impact Assessment (2006), and Measures on the Disclosure of Environmental Information (Trial) (2008) mandate the disclosure of environmental information by governmental and corporate entities. These provisions address information asymmetry, reduce regulatory implementation costs, and enhance efficiency (37). Research highlights the effectiveness of these tools in reducing concentrations of CO_2_ and PM2.5 (38), fostering greater public involvement in environmental stewardship.

Despite the extensive research on environmental regulatory tools, limited attention has been given to the role of environmental courts—an essential innovation in professionalizing environmental justice—in influencing individual health outcomes. As judicial bodies dedicated to adjudicating environmental disputes, environmental courts play a pivotal role in promoting environmental protection and safeguarding public health rights. Their rulings and judgments exert a substantial impact on mitigating pollution and protecting vulnerable populations. Building on existing studies, this paper seeks to bridge the gap by focusing on the relationship between environmental justice, as facilitated by environmental courts, and the health outcomes of older adults. Through this lens, the study aims to provide valuable insights into how specialized environmental judicial mechanisms can contribute to addressing the dual challenges of environmental governance and public health protection.

### 2.2 Institutional background

**Figure 1:**
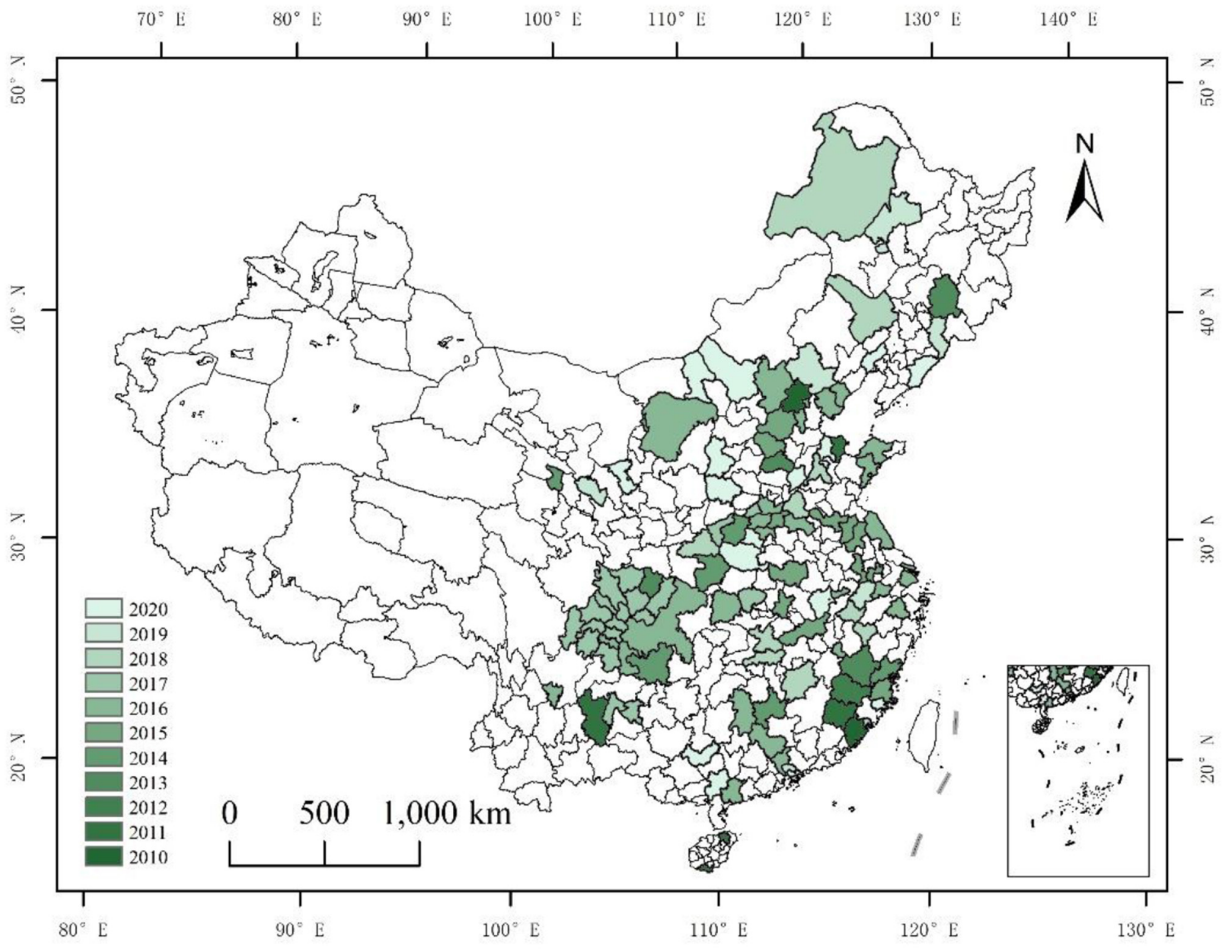
Map of pilot cities.

China's pilot environmental court policy holds significant contemporary relevance. Since the reform and opening-up period, China's economy has experienced rapid growth, but this progress has been accompanied by a surge in environmental violations by enterprises (39). These violations not only disrupt ecological balance and threaten public health but also expose critical shortcomings in China's environmental legal framework (40). Despite the introduction and implementation of numerous environmental laws, regulations, and administrative ordinances—including the two iterations of the Environmental Protection Law of the People's Republic of China (41)—the inherent complexities of environmental resource cases present unique challenges. These challenges include the multiplicity of litigation relationships, lengthy and costly assessment, and appraisal cycles, difficulties in preserving evidence, intricate causation chains, and the limitations of adjudicating environmental disputes through traditional courts (42). As a result, only a fraction of environmental disputes are effectively resolved through the existing justice system, leading to inefficiencies in enforcing environmental legislation and significantly undermining its effectiveness. In this context, China's traditional environmental justice system has proven inadequate in addressing the increasing prevalence of pollution cases, necessitating the establishment of environmental tribunals. Environmental tribunals are pivotal in achieving the specialization of environmental justice. The world's first environmental tribunal was established in Australia in 1980, followed by similar institutions in New Zealand, Sweden, and over 40 other countries (43). In December 2007, under the authorization of the Supreme People's Court (SPC), China's first specialized environmental tribunal—the Qingzhen Environmental Resources Tribunal—was established in Qingzhen, Guizhou Province, in response to a pollution case involving Tianfeng Chemical's discharge of wastewater. This marked the beginning of China's journey toward the specialization of environmental justice. In 2008, the Taihu Lake cyanobacteria crisis spurred the establishment of the Wuxi Municipal Intermediate Court Environmental Court, further advancing the reform of environmental judicial specialization. In 2011, Hainan Province set up the Higher People's Court Environmental Trial Court. The systematic reform reached a new milestone in June 2014, when the SPC established its own environmental tribunal, catalyzing the creation of specialized environmental judicial institutions nationwide (40). By the end of 2020, China had set up a total of 1,993 specialized judicial institutions for environmental resources, including 617 environmental resource trial courts, 1,167 collegiate panels, and 209 circuit courts, forming a comprehensive system for specialized environmental trials (44). Figure 1 illustrates the geographic distribution of pilot cities for environmental courts, which form the basis of this study. The research takes the environmental court pilot as a quasi-experiment, with the policy pilot period spanning from 2011 to 2020. The pilot areas cover China's eastern, central, and western regions, providing a robust experimental environment for applying a multi-period Difference-in-Differences (DID) model.

## 3 Empirical research design

### 3.1 Data sources

There are two main sources of data for this study: the China Health and Retirement Longitudinal Study (CHARLS) database, a large-scale interdisciplinary survey project hosted by the Institute of National Studies at Peking University and executed by the China Social Science Research Centre. It was conducted on a random basis and surveyed households and individuals of middle-aged and older adults ≥45 in China in 2011 on various aspects of information, including basic information, health status, and healthcare, to complete the collection of baseline data, and conducted four follow-up surveys in 2013, 2015, 2018, and 2020. Up to now, CHARLS has been conducted in 150 counties and 450 communities (villages) in 28 provinces (autonomous regions and municipalities directly under the central government) nationwide, covering 19,000 respondents in 12,400 households. Based on the CHARLS database, this paper selects five periods of data from the establishment of the database in 2011 to 2020 for empirical analysis. The second is the China Regional Statistical Yearbook (CRSY), an informative annual publication compiled and printed by the National Bureau of Statistics (NBS) that comprehensively reflects the economic and social development of China's regions, and the data for the city-level control variables involved in this paper come from this document.

### 3.2 Variable construction

#### 3.2.1 Core explanatory variable-environmental justice (EJ)

This article obtains the specific years of setting up environmental courts in prefecture-level cities from the websites of the intermediate people's courts of various prefecture-level cities, and constructs the 0–1 virtual variable of environmental courts as agent variables of environmental justice. If the prefecture-level city begins or has established an environmental court in that year, the value will be 1, otherwise it will be 0.

#### 3.2.2 Dependent variable-health status of the older adults (HSE)

The dependent variable of older adults' health in this study consists of two dimensions: physiological health and mental health.

Physiological health considers acute and chronic diseases and self-assessment health scores in older individuals as indicators. At the level of acute and chronic diseases, we follow the practices of Wang et al. (45, 46) to count acute and chronic diseases confirmed by doctors, including heart disease, stroke, cancer, and chronic diseases, including COPD, asthma, hypertension, dyslipidemia, diabetes, liver disease, kidney disease, stomach disease, and arthritis. Each confirmed type is 1 point, and the two items are added together to get a total score of 0–12 points. The higher the score, the worse the physiological health status. At the same time, self-assessment health scores for the older adults were introduced. According to the personal selection results in the survey question “How do you think you are healthy?,” we assign a value of 1 to those who choose “very bad,” a value of 2 to those who choose “bad,” a value of 3 to those who choose “general,” a value of 4 to those who choose “good,” and a value of 5 to those who choose “very good.” At the psychological level, we adopted the research ideas of Wen et al. (47) and used two indicators: cognitive function and the CES-D-10 self-evaluation scale. The multi-layered nature of Wen et al. can overcome the one-sidedness of a single depression or a single cognitive indicator. According to the practices of Wang et al. (45), this paper uses the entropy weight method to synthesize physiological and mental health indicators into the comprehensive index of older adults' health HSE to reflect the overall health status. The definitions and attributes of indicators at each level are shown in Table 1.

**Table 1:** Index definition and properties of HSE.

**Level 1 indicator**	**Level 2 indicator**	**Index explanation**	**Attributes**
Physiological health	Acute and chronic diseases	Including acute diseases such as heart disease, stroke, cancer, and chronic diseases such as pulmonary diseases, asthma, hypertension, dyslipidemia, diabetes, liver disease, kidney disease, stomach disease, arthritis, etc., one point is given for each disease. Score interval: [0, 12]	Negative
	Self-evaluation of health	According to the personal selection results in the survey question “How do you think you are healthy?,” select “Very bad” assign a value of 1, assign a value of 2 to those who choose “bad” as well, select “General” as 3, select “Good” as 4, and select “Very good” as 5. Score interval: [0, 5]	Positive
Mental Health	Cognitive function	It includes calculations, inquiry of dates, seasons, drawings and other questions. The number of times the respondents is correct is the psychological cognitive score. Score interval: [0, 12]	Positive
	Self-evaluation of depression	There are 10 questions involving the respondents' feelings and behaviors last week. The respondents choose from four options that indicate the frequency of occurrence. Adding the scores represented by the options is a self-rated score for depression. Score interval: [0, 30]	Negative

#### 3.2.3 Control variables

This includes personal characteristics of older persons and characteristics of the regional environment. In order to accurately capture the impact of environmental justice on the health status of rural older adults and to reduce endogeneity, it is necessary to introduce some control variables. The health status of rural older people is jointly affected by a variety of factors, which are analyzed in this paper in terms of both personal characteristics and regional environmental characteristics. Firstly, from the perspective of personal characteristics of the older adults, with the increase of age, the physical function of the older adults gradually declines, immunity decreases, and the risk of disease increases. Therefore, age is an important factor affecting the health of the older adults. Meanwhile, there are significant differences in health status and disease risk by gender (Gender). Even if men and women are equally exposed to the same risk or disease, the health consequences may be different for each gender (48). The health consequences for each sex may be different. Education (Edu) has a significant impact on the level of health prevention among older people, with those with higher levels of education often having better health knowledge and health behaviors and being able to prevent disease more effectively (48). Older adults are typically married, unmarried, divorced, or widowed, and studies have shown that marital status affects the level of social support and mental health status of older adults (49). Health-related behaviors include whether or not they smoke and whether or not they have a smoking habit. Health-related behaviors include smoking and alcohol consumption. Numerous studies have shown that smoking (Smoken) and drinking (Drunk) are important risk factors for a variety of diseases such as chronic lung disease, hypertension, cirrhosis of the liver, etc., and are negatively associated with overall health. In addition, higher economic income is usually associated with better living conditions and medical care, thus contributing to the overall health of older persons.

Variables measuring regional environmental characteristics include the gross regional product (lnGDP), the share of secondary industry (SSI), the number of people per million square kilometers (lnMIDU), medical level (ML), centralized treatment rate of wastewater treatment plants (CTRS), non-hazardous domestic waste disposal rate (NDWDR), and green coverage of built-up areas (GCBA). A higher GDP usually reflects a prosperous regional economy that can provide better public services and healthcare resources, thus helping to safeguard health in old age. Regions with a high share of secondary industries may have more air pollution, which is prone to negatively affect the health of the older population (50). Higher healthcare staff ratios tend to imply better primary healthcare systems and better access to healthcare services, which are strongly associated with healthier older people. The descriptive statistics of the data are shown in Table 2. A total of 23,895 rural older adults participated in this survey.

**Table 2:** Descriptive statistics for the main variables.

**Variable**	**Obs**	**Mean**	**Std. Dev**.	**Min**	**Max**
HSE	23,895	0.541	0.169	0.005	0.975
EJ	23,895	0.537	0.499	0	1
Gender	23,895	0.479	0.5	0	1
Age	23,895	68.742	6.618	60	120
Edu	23,895	1.817	1.01	1	4
Marry	23,895	2.224	2.381	1	8
Smoken	23,895	.256	0.436	0	1
Drunk	23,895	.313	0.464	0	1
Income	23,895	9.366	1.611	0	14.859
lnGDP	23,895	16.868	0.847	14.395	19.438
SSI	23,895	3.754	0.235	2.56	4.264
lnMIDU	23,895	8.092	0.655	6.317	9.619
ML	23,895	4.235	0.23	3.624	4.652
CTRS	23,895	90.242	11.683	24.03	99.996
NDWDR	23,895	96.215	11.011	11.61	100
GCBA	23,895	40.467	4.643	19.63	57.1

### 3.3 Empirical model

The Difference-in-Differences (DID) model, widely employed in policy evaluation, assesses the effects of a policy by comparing the changes in outcomes before and after the policy intervention between the treatment group (policy intervention group), and the control group. This methodology is particularly suited for this study, as the implementation of environmental justice policies provides a quasi-natural experimental setting (51). The DID model combines “before-and-after differences” with “between-group differences,” allowing it to control for the influence of confounding factors unrelated to the intervention. Additionally, the inclusion of covariates that may affect the outcome variables helps account for “suspected” influences, addressing the limitations of natural experiments, such as the inability to achieve completely randomized sample allocation. In this study, the DID model is utilized to empirically examine the impact of environmental justice, as represented by the establishment of environmental courts, on the health outcomes of older adults. The model is specified as follows:


(1) $$\begin{array}{l} HSE_{it}=\alpha_{0}+\alpha_{1}EJ_{it}+\sum \alpha_{2}Control_{it}+\gamma_{i}+\mu_{t}+\;\varepsilon_{it} \end{array}$$

In this model, the *HSE*_*it*_ represents the health level of the older person at time t, and EJ is the core explanatory variable. Drawing on Fan Ziying's study, this paper obtains the specific year in which the environmental court is set up in each prefecture from the website of the Intermediate People's Court of each prefecture. It constructs a 0–1 dummy variable to serve as a proxy for environmental justice, which takes the value of 1 if the older person's place of residence is set up or has been set up in the year t. Otherwise, it takes the value of 0. The variable corresponds to the interaction term in the traditional double-differential approach. *Control*_*it*_ represents control variables for a range of personal and regional environmental characteristics of rural older people, with γ_*i*_ as regional fixed effects and μ_*t*_ as year fixed effects.

## 4 Empirical results and analysis

### 4.1 Benchmark regression results

The impact of air pollution control policies, represented by environmental justice, on the health of the older adults, was analyzed, and the baseline regression results are summarized in Table 3. Column (1) in Table 3 presents the estimation results without incorporating any control variables. The coefficient for environmental justice (EJ) is significantly positive at the 1% level, indicating that the implementation of environmental justice policies is associated with improvements in the health status of older adults. Column (2) includes both individual characteristic variables (e.g., age, education level, marital status, gender, income, smoking habits, and alcohol consumption), and regional environmental characteristic variables [e.g., gross regional product (lnGDP), the share of secondary industry (SSI), medical care level (ML), centralized treatment rate of sewage treatment plants (CTRS), non-hazardous domestic waste disposal rate (NDWDR), and green coverage of built-up areas (GCBA)]. The inclusion of these variables enhances the model's explanatory power, as indicated by an increase in the model's goodness of fit. Notably, the EJ coefficient remains significantly positive at the 1% level, confirming the robustness of the baseline regression and highlighting the consistently positive impact of environmental justice policies on older adults' health.

**Table 3:** Benchmark regression results.

**Variable**	**(1)**	**(2)**
	**HSE**	**HSE**
EJ	0.0213^***^	0.0173^***^
	(0.0068)	(0.0059)
Gender		0.0455^***^
		(0.0023)
Age		−0.0034^***^
		(0.0001)
Edu		0.0541^***^
		(0.0011)
Marry		−0.0023^***^
		(0.0004)
Smoken		−0.0009
		(0.0024)
Drunk		0.0294^***^
		(0.0022)
Income		0.0141^***^
		(0.0007)
lnGDP		−0.0165^**^
		(0.0082)
SSI		−0.0124
		(0.0134)
lnMIDU		0.0092^**^
		(0.0044)
ML		0.0254^*^
		(0.0153)
CTRS		−0.0003^**^
		(0.0001)
NDWDR		0.0001
		(0.0001)
GCBA		0.0003
		(0.0004)
_cons	0.5300^***^	0.6567^***^
	(0.0038)	(0.1167)
*N*	23,895	23,895
*R* ^2^	0.097	0.329

Standard errors in parentheses.

* *p* < 0.1,

** *p* < 0.05,

*** *p* < 0.01.

### 4.2 Heterogeneity tests

#### 4.2.1 Geographical distribution

The implementation of environmental judicial policies needs to be adapted to local conditions. The Yangtze River and Yellow River Basins are not only the densest population and most concentrated industries in my country, but also the “pollution corridor” with the highest environmental risks in the country (52). High-density pollution exposure makes the older population one of the most vulnerable victims. Because of this, the 14th Five-Year Plan lists the Yangtze River and the Yellow River as the strategic main battlefields for water ecological environment protection (53). The environmental court density of cities along the main stream of the Yangtze River and the Yellow River was significantly higher than that of the Pearl River Basin and Songhua River Basin (54, 55). In addition, the climate, hydrology and industrial structure inside the basin are relatively homogeneous and more comparable, which can effectively reduce estimation errors caused by unobservable regional differences. In contrast, the traditional “East-China-Western” division reflects more the economic development gradient than the differences in pollution exposure, and it is difficult to focus on the causal logic of the role of environmental justice on health. Therefore, this study takes the Yangtze River and Yellow River Basins as analysis units, which not only remains consistent with the national major basin governance strategy, but also improves the policy pertinence of the conclusions. In Table 4, the samples of older people in cities along the Yangtze River and Yellow River Basin and cities along the non-Yangtze River Basin were respectively analyzed. The results show that the coefficient of the regression results of the older adults in cities along the Yangtze River and Yellow River Basin was 0.0191, which was significant at the confidence level of 1%. The coefficient of the regression results of the older adults in cities along the non-Yangtze River and Yellow River Basin was 0.0138, which was not significant at the confidence level of 10%, indicating that the implementation and development of environmental judicial policies have more outstanding effects on the improvement of the physical health status of the older adults in cities along the Yangtze River and Yellow River Basin.

**Table 4:** Geographical distribution heterogeneity test.

**Variable**	**Cities along the Yangtze and Yellow river basins**	**Non-route cities**
	**HSE**	**HSE**
EJ	0.0191^***^	0.0138
	(0.0070)	(0.0114)
Gender	0.0496^***^	0.0328^***^
	(0.0026)	(0.0047)
Age	−0.0035^***^	−0.0032^***^
	(0.0002)	(0.0003)
Edu	0.0563^***^	0.0479^***^
	(0.0012)	(0.0021)
Marry	−0.0022^***^	−0.0026^***^
	(0.0005)	(0.0009)
Smoken	−0.0006	−0.0030
	(0.0028)	(0.0047)
Drunk	0.0287^***^	0.0321^***^
	(0.0025)	(0.0046)
Income	0.0140^***^	0.0141^***^
	(0.0008)	(0.0013)
lnGDP	−0.0026	−0.0346^**^
	(0.0124)	(0.0155)
SSI	−0.0280^*^	0.0174
	(0.0163)	(0.0257)
lnMIDU	0.0117^**^	0.0107
	(0.0050)	(0.0119)
ML	0.0054	0.0071
	(0.0249)	(0.0322)
CTRS	−0.0005^***^	0.0001
	(0.0002)	(0.0003)
NDWDR	0.0001	0.0002
	(0.0001)	(0.0003)
GCBA	0.0003	0.0002
	(0.0005)	(0.0007)
_cons	0.5602^***^	0.9044^***^
	(0.1855)	(0.2785)
*N*	17,923	5,972
*R* ^2^	0.344	0.276

Standard errors in parentheses.

* *p* < 0.1,

** *p* < 0.05,

*** *p* < 0.01.

#### 4.2.2 Educational attainment (Edu)

Research has shown that education has a significant effect on the daily behavioral competence and mental health of older people. Older people with higher levels of education have better daily behavioral competence and mental health status (56). Therefore, this study suggests that the effect of the level of development of environmental justice on the physical health status of older adults may be heterogeneous depending on their level of education. In this test of heterogeneity, it was set that older adults with primary education and below were the low education group, and junior high school and above were the high education group. The results in Table 5 reveal that for the low education group, the coefficient is 0.0173, significant at the 5% confidence level, indicating that environmental justice policies have a stronger positive impact on the health of less-educated older individuals. While for the high education group, the coefficient is 0.0134, which is not significant at the 10% level, suggesting a weaker or negligible effect. These findings highlight that individuals with lower educational attainment are more sensitive to improvements in environmental conditions facilitated by justice policies.

**Table 5:** Educational attainment heterogeneity test.

**Variable**	**Higher academic qualifications**	**Lower academic qualifications**
	**HSE**	**HSE**
EJ	0.0134	0.0173^**^
	(0.0120)	(0.0068)
Gender	0.0062	0.0512^***^
	(0.0042)	(0.0028)
Age	−0.0030^***^	−0.0034^***^
	(0.0003)	(0.0002)
Edu	0.0181^***^	0.0823^***^
	(0.0036)	(0.0025)
Marry	−0.0007	−0.0024^***^
	(0.0009)	(0.0005)
Smoken	−0.0077^*^	0.0005
	(0.0040)	(0.0029)
Drunk	0.0311^***^	0.0300^***^
	(0.0036)	(0.0026)
Income	0.0179^***^	0.0135^***^
	(0.0014)	(0.0008)
lnGDP	0.0184	−0.0332^***^
	(0.0133)	(0.0101)
SSI	−0.0432^*^	−0.0008
	(0.0245)	(0.0160)
lnMIDU	0.0043	0.0117^**^
	(0.0087)	(0.0051)
ML	0.0325	0.0284
	(0.0291)	(0.0179)
CTRS	−0.0002	−0.0004^**^
	(0.0003)	(0.0002)
NDWDR	0.0001	0.0001
	(0.0002)	(0.0001)
GCBA	0.0015^*^	0.0001
	(0.0008)	(0.0005)
_cons	0.2106	0.8436^***^
	(0.2047)	(0.1415)
*N*	5,924	17,971
*R* ^2^	0.140	0.254

Standard errors in parentheses.

* *p* < 0.1,

** *p* < 0.05,

*** *p* < 0.01.

#### 4.2.3 Types of cities

Resource-based cities, where the economy is predominantly reliant on the extraction and processing of natural resources, present unique challenges. While economic development and urbanization in these cities may bring positive health outcomes, they are often offset by the adverse effects of environmental degradation. Optimizing resource allocation, improving environmental quality, increasing healthcare access, and promoting health education are essential strategies for mitigating these negative impacts. To examine the heterogeneity in the impact of environmental justice policies across city types, regression analyses were conducted for resource-based and non-resource cities, as shown in Table 6. For non-resource cities, the coefficient is 0.0192, which is significant at the 1% confidence level, indicating a robust positive effect of environmental justice policies on older adults' health. For resource-based cities, the coefficient is 0.0133, which is not significant at the 10% level, suggesting a weaker impact. These results suggest that environmental justice policies are more effective in improving health outcomes for the older adults in non-resource cities.

**Table 6:** Types of cities heterogeneity test.

**Variable**	**Non-resource-based cities**	**Resource-based city**
	**HSE**	**HSE**
EJ	0.0192^***^	0.0133
	(0.0072)	(0.0104)
Gender	0.0441^***^	0.0475^***^
	(0.0029)	(0.0037)
Age	−0.0034^***^	−0.0034^***^
	(0.0002)	(0.0002)
Edu	0.0542^***^	0.0538^***^
	(0.0013)	(0.0018)
Marry	−0.0022^***^	−0.0023^***^
	(0.0005)	(0.0007)
Smoken	−0.0033	0.0028
	(0.0031)	(0.0039)
Drunk	0.0290^***^	0.0304^***^
	(0.0028)	(0.0035)
Income	0.0142^***^	0.0140^***^
	(0.0008)	(0.0011)
lnGDP	−0.0058	−0.0256^**^
	(0.0115)	(0.0127)
SSI	−0.0275	0.0079
	(0.0183)	(0.0206)
lnMIDU	0.0037	0.0236^**^
	(0.0052)	(0.0097)
ML	0.0121	0.0427
	(0.0187)	(0.0310)
CTRS	−0.0003	−0.0003
	(0.0002)	(0.0002)
NDWDR	0.0003	0.0000
	(0.0002)	(0.0002)
GCBA	0.0004	0.0004
	(0.0006)	(0.0006)
_cons	0.6170^***^	0.5364^***^
	(0.1606)	(0.1888)
*N*	14,313	9,582
*R* ^2^	0.323	0.330

Standard errors in parentheses.

* *p* < 0.1,

** *p* < 0.05,

*** *p* < 0.01.

### 4.3 Robustness tests

#### 4.3.1 Parallel trends test

**Figure 2:**
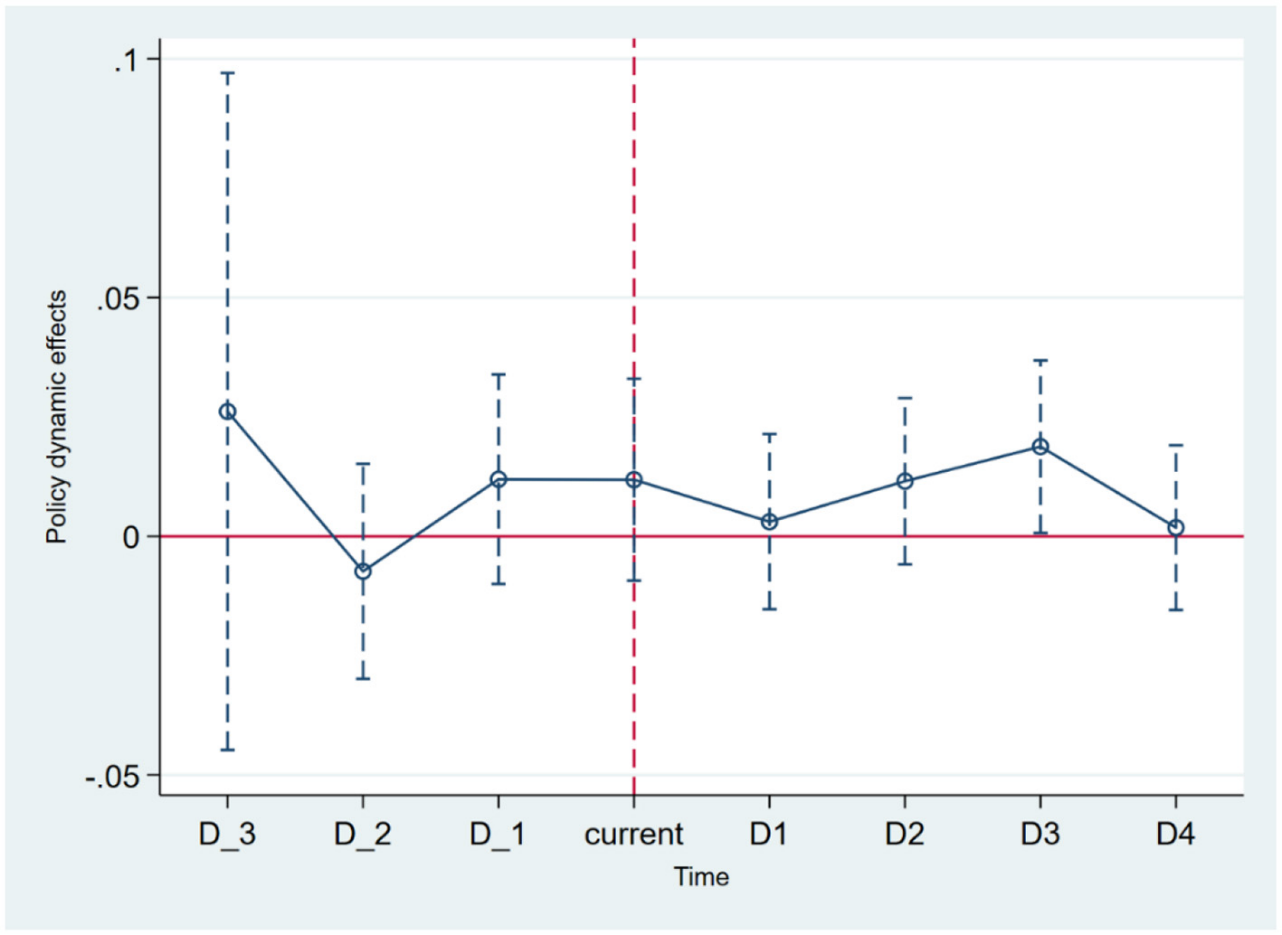
Parallel trend test.

The double-difference-in-differences (DID) methodology assumes a parallel trend between the treatment (pilot) and control (non-pilot) groups prior to policy implementation. To test this assumption, the study uses the year of the policy's first implementation as the baseline. It examines trends 3 years before and 4 years after the introduction of the environmental justice policy. As illustrated in Figure 2, there are no significant differences between the health trend lines of the pilot and non-pilot cities prior to policy implementation. This confirms that the parallel trend assumption holds, providing validity to the DID model.

#### 4.3.2 Exclusion of provincial capital cities

Provincial capital cities often have distinct characteristics, including better infrastructure, higher administrative capacity, and more comprehensive resources, which may influence the robustness of the regression model. To address this potential bias, the study conducts a regression analysis excluding provincial capital cities. The findings presented in Table 7 show that the coefficient of the core explanatory variable (EJ) remains significantly positive. This indicates that the benchmark regression results are robust, even after excluding provincial capital cities.

**Table 7:** Exclusion of provincial capital cities test.

**Variable**	**Excluding provincial capitals**
	**HSE**
EJ	0.0141^**^
	(0.0064)
Gender	0.0484^***^
	(0.0025)
Age	−0.0033^***^
	(0.0002)
Edu	0.0543^***^
	(0.0012)
Marry	−0.0024^***^
	(0.0004)
Smoken	−0.0001
	(0.0026)
Drunk	0.0291^***^
	(0.0023)
Income	0.0139^***^
	(0.0007)
lnGDP	−0.0290^***^
	(0.0088)
SSI	0.0094
	(0.0144)
lnMIDU	0.0110^**^
	(0.0047)
ML	0.0193
	(0.0169)
CTRS	−0.0002^*^
	(0.0001)
NDWDR	0.0001
	(0.0001)
GCBA	0.0002
	(0.0004)
_cons	0.7886^***^
	(0.1217)
*N*	20,870
*R* ^2^	0.328

Standard errors in parentheses.

* *p* < 0.1,

** *p* < 0.05,

*** *p* < 0.01.

#### 4.3.3 PSM-DID

To address potential self-selection bias, this study employs a propensity score matching double-difference (PSM-DID) approach. The 1:1 nearest-neighbor matching method is used, with older adults' characteristics from the control variables as the matching variables. This method reduces estimation bias by creating a balanced sample of treated and untreated individuals (57). The PSM-DID results in Table 8 confirm that the coefficient of EJ remains significantly positive, further supporting the robustness of the benchmark regression findings.

**Table 8:** PSM-DID 1:1 test.

**Variable**	**1:1**
	**HSE**
EJ	0.0177^***^
	(0.0059)
Gender	0.0456^***^
	(0.0023)
Age	−0.0034^***^
	(0.0001)
Edu	0.0539^***^
	(0.0011)
Marry	−0.0022^***^
	(0.0004)
Smoken	−0.0012
	(0.0024)
Drunk	0.0298^***^
	(0.0022)
Income	0.0140^***^
	(0.0007)
lnGDP	−0.0141^*^
	(0.0080)
SSI	−0.0111
	(0.0134)
lnMIDU	0.0293^*^
	(0.0152)
_cons	0.6689^***^
	(0.1151)
*N*	23,720
*R* ^2^	0.330

Standard errors in parentheses.

* *p* < 0.1,

** *p* < 0.05,

*** *p* < 0.01.

To enhance the utilization of the sample and improve the statistical efficacy of the study, a 1:4 nearest-neighbor matching method was employed. This method further alleviates self-selection issues and reduces estimation bias in the DID model. As shown in Table 9, the coefficient of EJ remains significantly positive, confirming the robustness of the findings across different matching methods.

**Table 9:** PSM-DID 1:4 test.

**Variable**	**1:4**
	**HSE**
EJ	0.0173^***^
	(0.0060)
Gender	0.0455^***^
	(0.0023)
Age	−0.0034^***^
	(0.0001)
Edu	0.0539^***^
	(0.0011)
Marry	−0.0022^***^
	(0.0004)
Smoken	−0.0010
	(0.0024)
Drunk	0.0299^***^
	(0.0022)
Income	0.0141^***^
	(0.0007)
lnGDP	−0.0145^*^
	(0.0081)
SSI	−0.0101
	(0.0135)
lnMIDU	0.0321^**^
	(0.0152)
_cons	0.6587^***^
	(0.1153)
*N*	23,611
*R* ^2^	0.330

Standard errors in parentheses.

* *p* < 0.1,

** *p* < 0.05,

*** *p* < 0.01.

### 4.4 Mechanism tests

**Figure 3:**
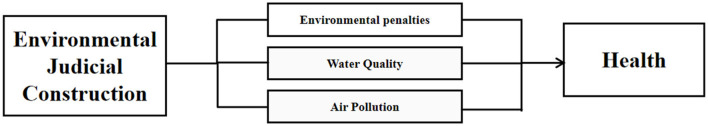
The mechanism framework of environmental judicial construction on older adults' health.

The benchmark regression results suggest that developments in environmental justice significantly enhance the physical health of older adults. Existing research underscores that air and water pollution have long been critical determinants of individual health (58). Air pollution contributes to elevated morbidity and mortality rates associated with respiratory diseases and cardiovascular conditions (16). Similarly, poor water quality exacerbates health risks, including cardiovascular diseases, mental health issues, and weakened bone health (59). Moreover, environmental penalties are regarded as an effective policy instrument for environmental protection, directly constraining the polluting behaviors of penalized firms (60). Accordingly, this study investigates the mechanisms through which environmental justice influences older adults' health, focusing on environmental penalties, water quality, and air pollution. The mechanism test framework is illustrated in Figure 3.

#### 4.4.1 Environmental penalties (EP)

Building on previous research, this study examines environmental penalties as a mediating variable to assess the impact of environmental justice on the physical health of urban older populations. The strength of environmental penalties is measured using the ratio of environmental penalty cases at the prefecture-level city to the local sulfur dioxide emissions. This data is sourced from the judicial case retrieval system of Peking University Fabulous, where a higher ratio indicates greater enforcement of environmental penalties. The regression coefficients in Table 10 are significantly positive at the 1% level, indicating that environmental justice policies significantly enhance the intensity of environmental penalties in areas where older adults reside. This, in turn, contributes to safeguarding their health by reducing environmental risks associated with industrial pollution.

**Table 10:** Environmental penalties mechanism test.

**Variable**	**(1)**
	**EP**
EJ	31.1450^***^
	(11.9029)
Pop	−0.0007
	(0.0034)
CTRS	−1.7269
	(1.1232)
NDWDR	−0.6827
	(0.5291)
GCBA	0.8770
	(0.5837)
_cons	91.4351
	(64.3209)
*N*	2,236
*R* ^2^	0.493

Standard errors in parentheses.

* p < 0.1,

** *p* < 0.05,

*** *p* < 0.01.

#### 4.4.2 Water quality (WQ)

Environmental justice provides a robust legal framework for the protection of water resources, addressing issues across multiple dimensions. In this study, a comprehensive water quality index is constructed using principal component analysis (PCA) to integrate indicators such as pH, dissolved oxygen (DO), permanganate index (CODMn), weekly water quality grades, and primary pollution indicators. The composite evaluation value serves as a proxy for water quality, where higher values indicate poorer water conditions. The regression coefficients in Table 11 are significantly negative at the 1% level. These findings demonstrate that environmental justice policies contribute to improving the health status of older adults by enhancing water resource quality and effectively reducing exposure to waterborne health risks.

**Table 11:** Water quality mechanism test.

**Variable**	**(1)**
	**WQ**
EJ	−0.2446^***^
	(0.0881)
Pop	−0.0000
	(0.0000)
CTRS	0.0070
	(0.0063)
NDWDR	−0.0003
	(0.0006)
GCBA	−0.0024^**^
	(0.0012)
_cons	2.7918^***^
	(0.2379)
*N*	1,344
*R* ^2^	0.644

Standard errors in parentheses.

* *p* < 0.1,

** *p* < 0.05,

*** *p* < 0.01.

#### 4.4.3 Air pollution (AP)

Environmental justice also plays a crucial role in addressing air pollution by remediating various environmental elements and suppressing pollutant emissions. This study evaluates air quality in older residential areas based on the concentration of pollutants such as PM2.5, a widely recognized indicator of air pollution. Higher pollutant concentrations reflect more severe air pollution. The regression coefficients in Table 12 are significantly negative at the 1% level, indicating that environmental justice policies are effective in mitigating air pollution. This improvement in air quality directly benefits the health of older adults by reducing respiratory and cardiovascular health risks associated with polluted air.

**Table 12:** Air pollution mechanism test.

**Variable**	**(1)**
	**AP**
EJ	−2.4917^***^
	(0.2498)
Pop	0.0001
	(0.0000)
CTRS	0.0422^***^
	(0.0158)
NDWDR	0.0014
	(0.0027)
GCBA	0.0056^**^
	(0.0024)
_cons	41.6863^***^
	(0.6561)
*N*	4,758
*R* ^2^	0.935

Standard errors in parentheses.

* *p* < 0.1,

** *p* < 0.05,

*** *p* < 0.01.

## 5 Discussion

### 5.1 Environmental justice policies are conducive to improving the health of the population

The benchmark regression results affirm that environmental justice policies significantly enhance the physical health of older people. This finding aligns with existing research, corroborating the notion that environmental regulation improves environmental quality and, consequently, public health outcomes (61). While prior studies have primarily focused on the impact of environmental legislation, this study extends the scope by integrating environmental justice into the research framework, offering a more comprehensive examination of its influence on the health of older adults (62).

The mechanisms driving the observed health benefits can be summarized as follows. Firstly, Environmental justice policies employ legal tools to regulate and penalize environmental pollution, thereby effectively curbing air, water, and soil pollution. By improving the quality of the living environment, these policies directly reduce older adults' exposure to hazardous environmental factors, enhancing their overall health (10, 63). Secondly, prolonged exposure to polluted environments heightens psychological stress among older adults, manifesting as anxiety, worry, and other negative emotions. Environmental justice policies, by improving environmental conditions, alleviate this stress and foster a more livable and tranquil environment conducive to better mental and physical health (25). In addition, the promotion of environmental justice policies encourages public participation in environmental governance. Increased public awareness leads to improved health literacy and the adoption of healthier behaviors, such as disease prevention and proactive health interventions. This not only enhances individual health but also strengthens community wellbeing through collective action (11, 64).

Despite their benefits, the implementation of environmental justice policies may also yield unintended consequences. Firstly, stringent regulations targeting resource-intensive industries can lead to significant short-term disruptions in production and lifestyle, imposing economic pressures on affected residents (65). Secondly, Limited information disclosure and immature regulatory mechanisms may cause residents to misunderstand policy intent or benefits, leading to resistance and psychological distress (66). In addition, in remote or resource-poor areas, weak economic foundations and inadequate infrastructure hinder effective policy implementation, potentially reducing the efficacy of environmental justice policies (67).

### 5.2 Discussion of heterogeneity

#### 5.2.1 The role of environmental justice policies in improving the physical health status of older people in cities along the Yangtze and Yellow River Basins

From the analysis of heterogeneous effects in Figure 3, it is evident that the impact of environmental justice policies on older adults' health is particularly pronounced in cities along the Yangtze and Yellow River Basins. As a national ecological security key protection area, the Yangtze River Basin enjoys a high degree of policy attention and support (68), and the deepened implementation and efficient promotion of environmental justice policies have significantly optimized the ecological environmental protection status of the basin. In addition, the Yangtze and Yellow River basins cover a vast geographical area, encompassing a large number of urban and rural areas, which are closely related to the survival of coastal residents, and the national and local governments have invested a large number of resources in the environmental management and public health protection of these areas, and implemented a series of stringent environmental protection (69). As a result, environmental justice reforms have helped to improve the quality of the environment in which older people live in the watersheds and to reduce the negative health impacts of environmental pollution, thereby significantly improving the health of older people. In other regions, where environmental issues have received less close attention from national and local policies, the effect of improving the physical health status of older adults has been relatively small.

#### 5.2.2 The role of environmental justice policies in improving the physical health status of older people with lower levels of education

The level of development of environmental justice policies has had a more significant impact on the health of older persons with lower levels of education. Older people with lower education levels usually lack sufficient awareness of health protection and concern for environmental issues. This cognitive limitation leads to a relatively disadvantaged state of health, especially in some areas where information is closed to the public. Due to the lack of environmental protection knowledge and awareness of the prevention of environmental pollution, older people with low education levels tend to live in poorer environmental conditions, which lead to a serious impact on their physical health. In addition, older people with low educational attainment often face lower income levels. When choosing their living environments, older people with lower incomes are often unable to avoid the environmental pollution and poor living conditions prevalent in low-income neighborhoods, further aggravating their health problems (70). Due to financial constraints, they may not be able to choose or move to areas with cleaner environments and have to put up with harsher environmental conditions, which directly affects their health (71). Therefore, environmental justice policies help to improve the living environment of low-income older people, and through health education and environmental protection knowledge dissemination, increase their awareness of environmental problems and preventive capacity, thus effectively reducing the health problems caused by environmental pollution.

#### 5.2.3 The role of environmental justice policies in improving the physical health status of older persons living in non-resource cities

The implementation of environmental justice policies has had a more significant effect on improving the health of older people living in non-resource cities. Resource-based cities are more resource-exploited than non-resource-based cities and often face more serious environmental pollution problems, especially in more industrialized areas where pollution pressures are heavier (72). When these cities do not have strict environmental regulations, older people face a higher risk of environmental pollution and public services tend to be more limited (73), which leads to a decreasing health level of local residents. In addition, numerous studies have shown that diseases caused by heavy environmental pollution are irreversible (73, 74). The implementation of environmental justice policies in resource cities has improved the living environment. However, the pollution problem has already caused irreversible health damage to a certain extent among the older adults. Therefore, because of their long-term dependence on resource extraction, the difficulty of pollution control and the slow process of ecological restoration in resource cities, this has led to a certain limitation in the effect of improving the health of older people, and the improvement of the health of older people living in resource cities as a result of environmental justice policies has not been significant despite the promotion of environmental justice policies.

## 6 Conclusions and policy recommendations

### 6.1 Conclusions

This paper explores the impact of environmental justice on the health of the older adults based on the CHARLS database with five periods of data from 2011 to 2020, using double-difference-in-differences (DID) model. The main conclusions are as follows: (1) Environmental justice policies can help improve the health of the older adults. (2) The heterogeneity study shows that, firstly, the implementation and development of environmental justice policies have a more prominent effect on the improvement of the health status of the older adults in the cities along the Yangtze River and Yellow River Basin. Secondly, the level of development of environmental justice has a more obvious effect on the improvement of the health status of the older adults with a lower level of educational attainment. Lastly, the level of development of environmental justice has a more obvious effect on the improvement of the health status of the older adults who reside in the non-resource-based cities. (3) The analysis of the mechanism shows that environmental justice has strengthened environmental penalties, improved water quality and reduced air pollution in the places where the older adults live, thus promoting the health of the older adults.

### 6.2 Policy recommendations

Based on the findings of the study on the impact of the establishment of environmental tribunals on the level of health of the older adults, future policy formulation and implementation should focus on the following areas:

(1) Accelerating the establishment of environmental courts and promoting environmental justice. The establishment of environmental courts has significantly improved the health of older persons, demonstrating the importance of expediting the establishment of environmental courts and promoting environmental justice. Therefore, the government should continue to speed up the establishment of environmental courts and develop a more comprehensive environmental justice system to ensure that environmental governance behaviors can play their proper roles in order to protect the health of older persons.

(2) Focus on regional differences and population differences and implement differentiated policies. Research shows that environmental courts play a more prominent role in improving the health of the older adults in cities along the Yangtze River and Yellow River Basin and non-resource cities, so the government should focus on environmental governance in cities along the Yangtze River and Yellow River Basin, consider the urban differences, formulate corresponding measures for the characteristics of different cities, and take stronger measures to improve the quality of water and air; given that the level of development of environmental justice has a more obvious role in improving the physical health of the older adults living in non-resource cities, the government should optimize the environmental governance strategy in non-resource cities, and formulate more effective measures for the characteristics of non-resource cities. Given that the level of development of environmental justice has a more obvious effect on the improvement of the physical health of the older adults living in non-resource cities, the government should optimize the environmental governance strategy of non-resource cities, and formulate a more effective environmental governance strategy that takes into account the characteristics of non-resource cities so as to promote the sustainable development of these cities. In addition, the development level of environmental justice has a more obvious effect on the improvement of the physical health of the older adults with lower educational attainment. Thus, the government should pay special attention to the health of the older group with low educational attainment and help them to improve their living environment and health by means of health education and the provision of medical assistance. By accurately assessing regional and population differences, differentiated policies should be implemented to more effectively improve the health of the older adults.

(3) Conducting publicity in the Environmental Court to raise public awareness of the environmental protection legal system. In addition to policy implementation, it is equally important to raise residents' awareness of the environmental justice system. The Government should, through publicity and educational activities, enhance residents' awareness of the environmental courts and encourage them to use the weapons of environmental justice to defend their reasonable rights and interests, so as to achieve synergistic development of environmental governance and public health and encourage the public to participate in environmental protection, so as to form a favorable atmosphere in which the whole society can pay attention to and support environmental justice.

(4) Deepening policy monitoring, evaluation and feedback mechanisms. With regard to the effectiveness of the implementation of environmental tribunals, a sound monitoring, evaluation, and feedback mechanism should be established. Changes in the health level of the population should be included as an important indicator in the evaluation of the policy, which should be continuously monitored and regularly evaluated, so as to make timely adjustments to and optimize the policy measures and to ensure that they are as effective as possible in promoting environmental governance and public health.

### 6.3 Limitations and future prospects

First, the CHARLS data used in this study relies on respondents' self-reported disease history, which may be biased from clinical diagnosis and is affected by memory decline and social expectations. Secondly, the older adults may experience spatial self-selective behavior of moving in or out in years when environmental quality is significantly improved or deteriorated due to the establishment of environmental courts, resulting in a dislocation between the level of residential exposure and the real long-term exposure, which in turn leads to spatial selection bias. Finally, future research can further include medical insurance claims big data, use objective medical results such as inpatient records, outpatient diagnosis and prescription information to reduce measurement errors, and use time-space matching with real-time concentrations of satellite remote sensing and ground monitoring to improve the accuracy of exposure assessment, thereby more comprehensively evaluating the long-term dividends established by environmental courts to the health of the older adults.

## Data Availability

The original contributions presented in the study are included in the article/supplementary material, further inquiries can be directed to the corresponding authors.
